# Lessons Learned after Iatrogenic Complete Transection of the Right Common Carotid Artery with Segmental Vessel Loss

**DOI:** 10.1155/2021/8812870

**Published:** 2021-03-27

**Authors:** Shamir O. Cawich, Wendell Dwarika, Fawwaz Mohammed, Michael J. Ramdass, Vindra Ragoonanan, Megan Augustus, Dave Harnanan, Vijay Naraynsingh, Richard Spence

**Affiliations:** ^1^Department of Surgery, Port of Spain General Hospital, West Indies, Trinidad and Tobago; ^2^Department of Surgery, Eric Williams Medical Sciences Complex, Trinidad and Tobago

## Abstract

Carotid arterial injuries occur in 5-6% of persons with penetrating trauma. Complete transection is rare in civilian practice and is most often due to penetrating injuries. Complete transection as an iatrogenic complication is rare. We present a case where we were required to repair a complete transection of the carotid artery with segmental loss which occurred as an iatrogenic complication during thyroidectomy. We could find no previous reports of this type of iatrogenic complication. The lessons learned during the management of this case were the following: (1) surgeons should call for help early, (2) a multidisciplinary approach ensures that all options are considered, (3) adhere to surgical principles of proximal and distal control, (4) always use atraumatic clamps to control vessels, and (5) flow restoration should be attempted, leaving carotid ligation as the last resort.

## 1. Introduction

Carotid artery (CA) injuries occur in 5-6% of persons with penetrating neck trauma [1, 2]. Injury patterns may vary from tangential lacerations with low-velocity trauma to complete transection from high-velocity projectiles. Complete transection is rare in civilian practice.

We recently managed a patient with a complete transection and segmental CA loss. There were no previous reports of this type of iatrogenic complication. We discuss the lessons learned.

## 2. Report of a Case

The surgical team on call, headed by a consultant general surgeon, was summoned to the operating room where a 79-year-old woman sustained CCA injury during thyroidectomy. A passing house officer attempted to control bleeding with hemostats for 60 minutes before requesting help.

**Figure 1:**
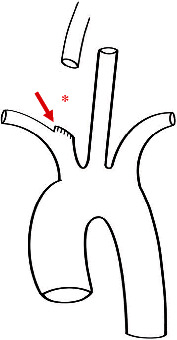
An illustration of the vascular injury and surrounding anatomy. The asterisk demonstrates the segmental loss of the carotid artery and the arrow points to the stub of proximal common carotid artery with multiple horizontal lacerations extending down to the bifurcation of the brachiocephalic trunk.

**Figure 2:**
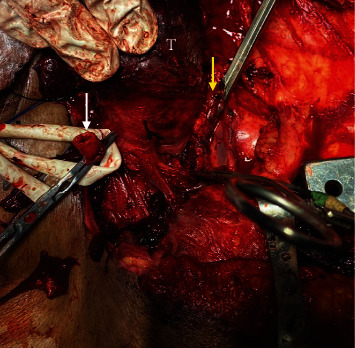
Intraoperative photograph detailing the operative site. The thyroid (T) is being retracted to expose the CA posteriorly. The transected distal CA is occluded with clamps (white arrow). The proximal CA is tenuously controlled with a clamp behind the sternoclavicular joint (yellow arrow).

In the operating field, we found a partially mobilized right thyroid lobe. The house officer completely excised a 6 cm segment of CA and applied hemostats to the transected ends. There was a stub of CA proximally (Figure 1) that bore multiple lacerations from uncontrolled hemostat applications (Figure 2). A median sternotomy was required to achieve proper control of the injured CA behind the sternoclavicular joint. At this point, temporary flow restoration was impossible because there was insufficient purchase of proximal carotid to place a vascular shunt. At this point, the CA had been clamped for 60 minutes and 1 L blood loss was already recorded.

**Figure 3:**
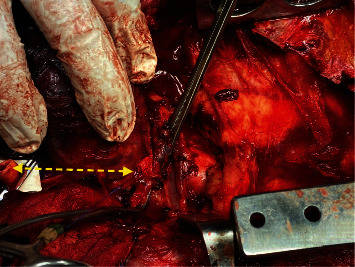
In this intraoperative photograph, the yellow arrows point to both ends of the CA and the broken line illustrates the segmental vessel loss.

A median sternotomy was rapidly performed using a previously described technique [3]. Proximal control was achieved when vascular clamps were applied to the right brachiocephalic trunk and subclavian artery. There was such a large segment loss that interposition grafting or transposition was required to restore continuity (Figure 3). Reverse interposition saphenous grafting was considered, in the absence of PTFE.

**Figure 4:**
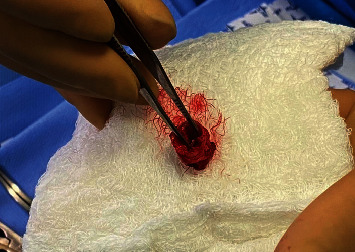
Photograph of the excised segment of proximal CA with multiple lacerations.

**Figure 5:**
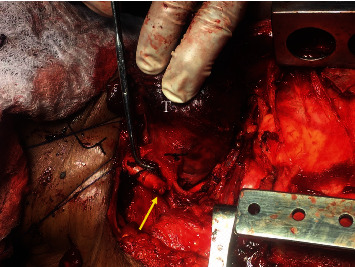
Intraoperative photograph demonstrating over sewn distal carotid artery with good hemostasis (yellow arrow). The ischemic right lobe of thyroid (T) is demonstrated in-situ before excision.

A brief time-out for multidisciplinary discussion between attending anesthetists and surgeons highlighted that there was now: absent cerebral perfusion for 150 minutes, 2 L blood loss, transient hypotension, and metabolic acidosis. There was consensus that the interposition grafting would not be worth the risk in an unstable and acidotic patient. Therefore, the proximal stump was excised (Figure 4) and both ends ligated (Figure 5).

She remained inotrope-dependent for 5 days and had a tracheostomy after 10 days. After sedation was discontinued, a left hemiparesis was observed and CT brain confirmed a right occipital lobe infarct. After a prolonged hospitalization and intense outpatient rehabilitation, there was full neurologic recovery as documented in the supplemental video that can be viewed using the attached link: https://onedrive.live.com/?authkey=%21AIvmiJK7lTTkrBk&cid=963EC1774FD012B0&id=963EC1774FD012B0%21990&parId=963EC1774FD012B0%21186&o=OneUp.

The right common CA originates at the bifurcation of the brachiocephalic trunk behind the sternoclavicular joint and courses cephalad, terminating at the level of the thyroid cartilage (C4 vertebral body) where it bifurcates into internal and external CA. It is at risk for injury during thyroidectomy. It may be prudent to involve vascular surgeons as a part of a multidisciplinary team approach when a high risk of injury is anticipated.

These injuries are particularly devastating because of cerebrovascular ischemia and airway compression. Multiple authors have documented high mortality after CA injuries in military practice as outlined in Table 1 [4–7]. Less severe injuries, and lower attendant mortality, have been reported in civilian practice [2, 8–24].

It is reasonable to expect that iatrogenic operative injuries would be even less severe because (1) its nature ensures a low energy injury, (2) the patient is already anesthetized, (3) the airway already controlled, (4) the injury is immediately identified due to bleeding, and (5) the neck is already exposed to facilitate repair. We performed a literature search evaluating iatrogenic CA injuries by searching Pubmed, Medline, EMBASE, and Google Scholar databases for the terms: iatrogenic, surgical, common carotid, internal carotid, transection, injury, penetrating, trauma, segmental loss, repair, and ligation. We only included patients with iatrogenic injuries at open neck surgery and excluded patients with iatrogenic needle or sheath injuries from percutaneous vessel catheterization, injuries at endoscopic nasal surgery, radiation-induced injuries, and chemical-related injuries, because the management of these injuries would be different. Our literature search returned a series of 22 cases published by Dorobisz et al. [25] between 1980 and 2003 and a series of 35 patients between 1999 and 2004 by Zhang et al. [26]. The data is presented in Table 2. Collectively, iatrogenic CA injuries were accompanied by 19.3% incidence of stroke and 10.5% mortality, lower than seen with penetrating trauma.

While it is clear that iatrogenic injuries, in general, are accompanied by less morbidity, it is also true that outcomes are affected by injury severity. Obviously, a patient with a tangential laceration would have a better outcome than one with transection with segmental loss. After a detailed literature search, we could not find another report of iatrogenic complete transection with segmental loss.

This case started as a tangential laceration, which should have been easy to manage when following surgical principles. A common error is for inexperienced surgeons to become flustered by arterial bleeding and use traumatic clamps to control the bleeding. This should be strongly discouraged as it can damage the delicate arterial walls and extend the injury. In our case, traumatic clamps extended the injury into the mediastinum and mandated a thoracotomy. The takeaway lesson is that inexperienced surgeons should call for appropriate help early.

After control with atraumatic vascular clamps, tangential lacerations should be repaired primarily using small caliber monofilament sutures with round body needles, taking care in suture placement to avoid raising intimal flaps. Although complete transections are usually followed by vessel retraction, a primary anastomosis without tension may still be achieved. With segment loss, the surgeon is now required to deal with (1) arterial retraction, (2) uncontrolled bleeding, (3) loss of continuity, (4) prolonged brain ischemia, and (5) possible interposition graft revascularization.

There are two options available: vessel ligation or flow restoration. Flow restoration brings the lowest rates of cerebrovascular ischemia [2, 15, 17, 19, 20] and can be achieved by primary end-to-end anastomosis, vein interposition, PTFE grafting, or arterial transposition [2, 24–26]. The choice of repair is dictated by the pattern of injury. End-to-end repairs are usually impossible when there is segmental loss because the anastomotic line would be under tension, leading to anastomotic blowout and/or pseudoaneurysm formation. Interposition grafting is often required and a vascular shunt should be considered while the interposition graft is prepared. The shunt provides cerebrovascular perfusion reducing the incidence of a stroke, but there is no clear consensus on its use [1, 21, 24,]. Most recommendations are extrapolated from carotid endarterectomy, and many authorities use selective shunting when dictated by EEG changes and occlusion tests [27–29]. In our case, shunting was not possible because proximal control could not be established.

Vein interposition is preferred for repairs with segmental loss [25]. But PTFE interposition avoids the need to dissect, harvest, and prepare vein grafts, thereby reducing ischemic time. Ugurlucan et al. [30] described the successful use of a prosthetic bypass between the left and right external CA in a patient with occlusion of the left subclavian and common CA. This might have been an option in our case, but proximal control and hemostasis would still have taken precedence.

Other options would have been carotid artery transposition. Bounds et al. [31] implanted one CA into the contralateral common CA. Transposition to ipsilateral subclavian [32, 33] or vertebral arteries [34] have also been described for occlusive disease and steal syndromes. However, the large segment loss would not have allowed a tension-free anastomosis and so these were not options in this case.

Ligation should be used as a last resort for damage control surgery, because it is associated with a high incidence of mortality and stroke [2, 7, 24, 25]. Most authorities agree that zone II injuries should only be ligated if there is persistent hypovolaemic shock, severe associated injury that independently predicts poor outcomes (e.g., brain gunshot wounds), multiple associated injuries that take priority to preserve life, or established ischemic infarcts [2, 7, 21]. Other indications for ligation (that are not relevant for surgical iatrogenic injuries) include severe cerebral oedema on preoperative CT, coma for 6 hours duration, patients with proven occlusion on angiography who have normal neurology [1, 2, 7]. Ligation was performed in our case because of the prolonged ischemia, persistent hypotension, severe metabolic acidosis, and the need for a thoracotomy for proximal control.

We reviewed the raw data from the studies above, specifically tabulating the outcomes of patients who underwent CA ligation where data was available (Table 3). Emergent ligation of the ipsilateral CA was accompanied by 21% mortality and 39% post-operative stroke rates. In this case, with little back bleeding from the ICA, we anticipated neurologic deficits in the postoperative period.

Over the past 2 decades, there has been a notable increase in the number of CA injuries managed via endovascular approaches [35–38]. However, with the large segmental loss, in this case, most endovascular approaches were not suitable. One endovascular maneuver described by Starnes and Arthurs [37] is worthy of consideration. They suggested the use of an endovascular balloon to create an intraluminal occlusion to control bleeding, allowing for controlled exploration [37]. This option was not considered in our case, and it could be argued that this may have averted the need for a thoracotomy to achieve control. A further argument could be made that control without a thoracotomy may have prompted us to reconsider arterial transposition. This highlights another important lesson: even in experienced hands, therapeutic options may be overlooked, reinforcing the need for communication and multidisciplinary team approaches.

## 3. Conclusion

Complete transections are uncommon as iatrogenic CA injuries. It is important to adhere to surgical principles of proximal and distal control, use of atraumatic clamps and properly selected sutures, early call for help, and multidisciplinary team approaches. Flow restoration is recommended either by primary anastomosis, interposition vein grafting, PTFE grafting, or arterial transposition. Modern endovascular techniques may also have a role in select cases.

## Figures and Tables

**Table 1:** Clinical outcomes after management of common/internal carotid arterial injury due to penetrating neck trauma.

Published study/data set	Total number of injuries	Deaths
Combat-related penetrating injuries:		
Rich et al., 1970 [4]	50	NS
Fox et al., 2005 [5]	2	NS
Fox et al., 2006 [6]	12	1/12 (8.3%)
Reva et al., 2011 [7]	46	13/46 (28.3%)
All combat-related CA injuries	**110**	**14/58 (24.1%)**
Civilian penetrating injuries:		
Cohen et al., 1970 [8]	85	13
Bradley et al., 1973 [9]	24	8
Rubio et al., 1974 [10]	71	17
Thal et al., 1974 [11]	60	5
Liekweg et al., 1978 [12]	18	4
Fry et al., 1980 [13]	54	5
Unger et al., 1980 [14]	464	97
Ledgerwood et al., 1980 [15]	36	12
Brown et al., 1982 [16]	129	27
Meyer et al., 1988 [17]	18	3
Richardson et al., 1988 [18]	33	2
Weaver et al., 1988 [19]	72	5
Demetriades et al., 1989 [1]	124	27
Fabian et al., 1990 [20]	35	12
Ramadan et al., 1995 [21]	55	12
Ditmars et al., 1997 [22]	11	0
Mittal et al., 2000 [23]	16	3
Navasaria et al., 2002 [2]	28	2
Du Toit et al., 2003 [24]	130	24
All civilian penetrating injuries	**1463**	**278/1463 (19%)**

**Table 2:** Clinical outcomes after management of common/internal carotid arterial injury from iatrogenic surgical trauma.

Published study/data set	Number of injuries	Deaths	Neurologic deficits/stroke
Dorobisz et al., 2005 [C]	22	2/22 (9%)	3/22 (13.6%)
Zhang et al., 2006 [D]	35	4/35 (11.4%)	8/35 (22.9%)
All iatrogenic injuries	**57**	**6/57 (10.5%)**	**11/57 (19.3%)**

**Table 3:** Clinical outcomes in patients undergoing CA ligation.

Study	Ligations	Deaths	Stroke
Liekweg et al., 1978 [12]	4	2	2
Ledgerwood et al., 1980 [15]	5	0	3
Meyer et al., 1988 [17]	2	0	0
Weaver et al., 1988 [19]	18	2	8
Fabian et al., 1990 [20]	6	2	4
Ramadan et al., 1995 [21]	17	0	8
Navasaria et al., 2002 [2]	3	1	2
Du Toit et al., 2003 [24]	20	9	2
Zhang et al., 2006 [D]	17	3	5
	**92**	**19 (20.7%)**	**34 (37.0%)**

## Data Availability

All data are stored by the corresponding author on an electronic database and can be made available upon request.
